# Validation of the Malta Gait Scale: A Time-Efficient Tool for Poststroke Assessment

**DOI:** 10.1155/srat/8849857

**Published:** 2025-03-27

**Authors:** Valerio Sarmati, Carlos Carmona, Alessandro Morciano, Samuel Gutiérrez, Ingrid Velásquez, José Fernández

**Affiliations:** ^1^Sapienza University of Rome, Rome, Italy; ^2^Stroke Therapy Revolution Ltd, Marsalforn, Malta; ^3^Hospital Virgen De Las Montañas, Villamartin, Andalucía, Spain; ^4^Universidad de Carabobo, Valencia, Carabobo, Venezuela

**Keywords:** Malta Gait Scale, observational gait analysis, physiotherapy practice, stroke rehabilitation, walking dysfunction

## Abstract

Over 80% of stroke survivors experience walking dysfunction, impacting quality of life. Rehabilitation is crucial for gait recovery, and accurate assessments facilitate tailored programs. While computerized gait analysis is the gold standard, it is costly and requires specialized training, making observational gait analysis (OGA) more common. However, OGA can also be time-consuming. This study validates the Malta Gait Scale (MGS), a concise, illustrated 7-item observational tool using video recordings for gait measurements. The aim is to provide an effective, time-efficient method for gait evaluations by comparing the MGS with the established Wisconsin Gait Scale (WGS) and Gait Assessment Intervention Tool (GAIT), which have 14 and 31 items, respectively. Forty-nine participants were included in a retrospective study to validate the MGS. We evaluated its reliability using weighted Cohen's kappa (*κ*) for intrarater and interrater reliability. Concurrent validity was assessed by comparing the MGS with the WGS and GAIT scales using Spearman's rho (*ρ*). The Wilcoxon test assessed the efficacy of the MGS in detecting rehabilitation-induced changes, differentiating healthy from stroke participants, and evaluating time efficiency. The MGS demonstrated almost perfect agreement, with interrater and intrarater *κ* values of 0.952 and 0.977, respectively. It showed high positive correlations with the WGS and GAIT, with *ρ* values of 0.898 and 0.877. MGS required an average administration time of 7 min and 29 s, significantly less than the WGS (27 min and 46 s) and GAIT (50 min and 6 s) (*p* < 0.001). Following rehabilitation, significant improvements were observed in patients using both the MGS and WGS scales (*p* = 0.018), and the MGS effectively distinguished between healthy individuals and stroke patients (*p* < 0.001). The MGS is a valid, reliable, and efficient tool for gait assessment in stroke survivors, supporting smartphone use and facilitating rapid measurements in clinical settings where time is critical.

## 1. Introduction

Stroke leads to an interruption of blood supply to the brain, depriving it of oxygen and causing significant health consequences [1]. It ranks as the second leading cause of death globally and is a major contributor to disability, affecting essential functions such as walking, grasping, and speaking [2–6]. Over 80% of stroke survivors experience walking dysfunction, which severely impacts their independence and quality of life [7, 8].

Rehabilitation that enhances cognitive, perceptual, and motor skills is crucial for gait recovery [9, 10]. Therefore, frequent and accurate assessments facilitate the customization of rehabilitation programs to suit the observed diverse and dynamic recovery patterns [11, 12].

Computerized gait analysis is acknowledged as the gold standard for providing precise and reliable measurements through advanced technological instruments [13, 14]. However, the high cost of equipment and the requirement for specialized training limit its widespread use in clinical settings, making observational gait analysis (OGA) the more commonly employed method for gait assessment [15–17].

OGA involves the visual observation of gait patterns by clinicians, with some implementations using video recordings of patient gait. These recordings afford observers the benefit of repeated viewings without the concern of inducing patient fatigue [15, 18]. Additionally, video recordings provide access to features beyond the capabilities of the naked eye, such as zoom, pause, and slow motion. While OGA is popular and straightforward, it offers less reliability when compared to computerized assessments [15, 19]. Despite its widespread use and simplicity, implementing OGA requires a significant investment of time for thorough observation. Among the tools for gait assessment in stroke patients that rely on video recordings for qualitative evaluations are the Wisconsin Gait Scale (WGS) with its 14 items [20] and the Gait Assessment Intervention Tool (GAIT) with 31 items [21].

Although the reliability and validity of each of these scales have been demonstrated [21–23], their use in daily rehabilitation routines might be limited due to the considerable time required for administration, a pivotal factor in the selection of assessment scales in the clinical environment [18]. This situation underscores the need for a gait analysis tool that is both efficient and conducive to evidence-based therapeutic decisions.

To address this need, our study validates the Malta Gait Scale (MGS), a concise 7-item tool for swift gait assessment. The aim of validating the MGS is to equip healthcare professionals with an effective option for conducting gait evaluations within the practical time constraints of daily practice. The MGS is designed to balance reliability with quick administration, facilitating informed treatment decisions in poststroke rehabilitation and meeting the clinical demand for accessible tools.

## 2. Materials and Methods

### 2.1. Participants

A total of 49 participants were included in this retrospective study, consisting of 25 males and 24 females, with a mean age of 59 ± 8.9 years. From 175 patients who had participated in a home-based rehabilitation program at Stroke Therapy Revolution Limited in Malta between January 1, 2016, and January 1, 2022, 41 stroke survivors were randomly selected, including 21 with left-side hemiparesis and 20 with right-side hemiparesis, based on the following inclusion criteria: (1) having suffered a brain stroke resulting in hemiparesis, (2) absence of any additional pathology impairing walking, (3) full independence in ambulation prior to the stroke, and (4) the ability to walk unaided by a person for at least 10 m. Within this group, seven individuals who had completed 4–6 months of rehabilitation were also randomly chosen to specifically evaluate the ability of the scale to detect changes in gait postrehabilitation. Additionally, eight healthy subjects were recruited as controls to assess the effectiveness of the scale in differentiating between healthy and poststroke gait patterns. All participants provided signed informed consent, and the study received approval from the ethics committee of Central Hospital in Maracay, Venezuela.

### 2.2. Observers

Thirty-six observers were involved in the study, including 31 physical therapists and five occupational therapists, with clinical experience ranging from 1 to 35 years. Their primary task was to review patient videos for assessment. It was noted that none of the observers had prior familiarity with the scales used in this study, ensuring an unbiased approach to the video evaluations.

### 2.3. Tools

The validation study of the MGS involved comparisons with two other scales: the WGS and the GAIT scale.

WGS: This scale comprises 14 items, with total scores ranging up to 42.25 points, indicating severe walking limitations in poststroke patients. A lower score signifies milder limitations, with the minimum score set at 13.35.

GAIT: It features 31 items, with scoring from 62 to 0. A higher score denotes more severe walking impairments, whereas a lower score indicates milder effects.

**Figure 1:**
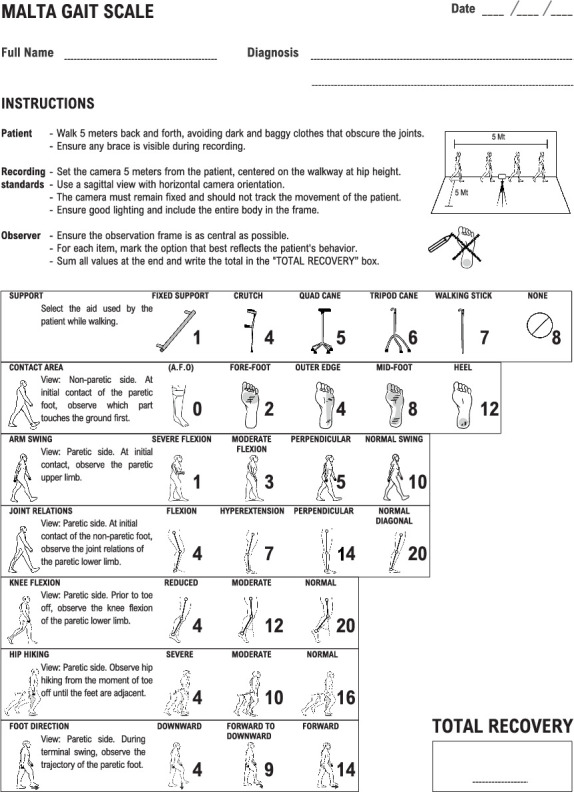
The Malta Gait Scale is used to assess gait in stroke survivors through seven observational items. Observations are made from video recordings, with specific instructions for each item regarding the view and the events of gait to be observed. The total recovery score is calculated by summing the marked values.

MGS: This scale includes seven items, with scores ranging from 18 to 100. A score of 18 indicates the most substantial gait deviations, while a score of 100 represents minimal gait deviations, signifying superior walking ability. Differently from WGS and GAIT, the MGS provides item descriptions alongside drawings that illustrate the engaged body segments in each phase of the gait, helping observers assess varying degrees of deviation (Figure 1).

### 2.4. Training

Each observer was sent a video tutorial for each scale, demonstrating the measurement tool with a video of a subject walking. This subject met the study's inclusion criteria but was not included in the list of participants.

### 2.5. Recording Standards

Participant gait was recorded according to the projection requirements specified by each scale. The MGS utilizes exclusively sagittal projections, whereas GAIT and WGS require additional sagittal, frontal, and posterior projections. Unlike GAIT and WGS, which do not provide detailed criteria for these recordings, MGS offers explicit recording guidelines. We adapted the detailed MGS recording standards to also cover the evaluations conducted with GAIT and WGS, respecting the required number of projections for each scale. This methodical approach ensured a standardized data collection process. Further specifics of the recording protocol are as follows:

Camera: It must remain stationary and not track the movements of participants. It should be placed 5 m from the walkway, aimed at its center, and adjusted to hip height for a consistent perspective.

View of the gait: The camera must be oriented horizontally with sufficient lighting ensured, and the camera operator must verify that all parts of the body are clearly visible on the screen.

Participants: Required to walk back and forth to display both sides. No specific instructions regarding walking speed are provided. Those who regularly wear an ankle-foot orthosis should keep it in view.

### 2.6. Equipment

All videos were recorded using ordinary commercially available smartphones of different brands with different operating systems, but all were capable of recording video with a minimum resolution of 7 megapixels and a minimum speed of 30 frames per second.

### 2.7. Intrarater Reliability and Interrater Reliability

The reliability of the MGS was evaluated with data from 34 stroke survivors and eight healthy subjects. From the pool of 36 observers in the study, 26 were randomly selected for this specific analysis. Intrarater reliability was determined by having observers assess the same videos twice, with a 14-day interval between assessments. Interrater reliability was assessed through comparisons of MGS scores assigned to each video by pairs of these observers. The consistency of the evaluations was quantified using the weighted Cohen's kappa (*κ*), which provided a measure of agreement [24]. Weighted *κ* values were interpreted using established standards. Specifically, values less than 0 represent *poor agreement*; between 0.01 and 0.20, *slight agreement*; between 0.21 and 0.40, *fair agreement*; between 0.41 and 0.60, *moderate agreement*; between 0.61 and 0.80, *substantial agreement*; and between 0.81 and 1.00, *almost perfect agreement* [25]. All analysis of the data was conducted using IBM SPSS, Version 29.0.2.0.

### 2.8. Concurrent Validity

To conduct the validation process, items from the MGS, WGS, and GAIT were categorized into “stance” and “swing” phases. Spearman's correlation coefficient (*ρ*) was then utilized to assess the relationships within these phases and across the total scores of the scales. Correlations were interpreted as follows: *negligible* (0.00–0.30), *low* (0.30–0.50), *moderate* (0.50–0.70), *high* (0.70–0.90), and *very high* (0.90–1.00) [26]. Statistical significance was determined with a *p* value of less than 0.05.

### 2.9. Efficacy of MGS in Detecting Rehabilitation-Induced Gait Changes

The study employed MGS and the WGS to assess changes in gait postrehabilitation among stroke survivors. From the initial cohort of 41 stroke survivors who had participated in a home-based rehabilitation program, seven individuals who met specific inclusion criteria were randomly selected for detailed analysis. These criteria ensured that only participants who had completed the rehabilitation program lasting between 4 and 6 months were included and who did not receive any other rehabilitative or surgical treatments during the examined period. The gait assessments were conducted at two-time points: baseline (T0) and after rehabilitation (T1), using the Wilcoxon test for statistical evaluation. Two observers, with respective clinical experiences of 4 and 19 years, were randomly selected from the group of 36 professionals to conduct the assessments.

### 2.10. Discrimination Between Healthy and Stroke Individuals

The ability of the MGS to distinguish between healthy individuals and stroke patients was evaluated using the Wilcoxon test. Data from eight healthy participants and 34 stroke patients were included in the analysis.

### 2.11. Analysis of Administration Time for Gait Scales

Observers recorded the time taken to administer each gait assessment scale to evaluate the efficiency of the MGS, WGS, and GAIT scales. A comparative analysis of average administration times was conducted. The coefficient of variation (CV) was calculated to assess the relative variability in administration times across the scales.

### 2.12. Usability Impact of Illustrations in MGS

To investigate the impact of illustrations on the MGS, we conducted a comparative analysis between the standard illustrated version and a newly developed text-only format. Twelve stroke patients and 16 observers, randomly selected from participants in the study, were divided into groups to assess either version of the scale. The study was aimed at exploring the effects of illustrations on assessment duration and interrater reliability. We employed the Wilcoxon test to analyze the assessment duration and Cohen's *κ* to assess interrater reliability.

## 3. Results

### 3.1. Reliability Assessments: Intra- and Interrater Findings

This section presents the reliability results for the MGS, as shown in Table 1. Interrater reliability exhibited weighted *κ* values ranging from 0.707 for hip hiking (substantial agreement) to 0.956 for support (almost perfect agreement), indicating varying degrees of consistency. Intrarater reliability showed *κ* values ranging from 0.856 for foot direction to 0.989 for support, both within the range of almost perfect agreement. The weighted *κ* values for the total scores of interrater and intrarater reliability were 0.952 and 0.977, respectively, both considered almost perfect agreement. The total scores for the other two scales used in this study also showed the following weighted *κ* values: 0.960 for interrater reliability and 0.983 for intrarater reliability in the WGS and 0.931 for interrater reliability and 0.984 for intrarater reliability in the GAIT scale.

### 3.2. Concurrent Validity Results

Table 2 illustrates that the MGS exhibited high positive correlations (*ρ* > 0.70) with both the WGS and GAIT across total scores, stance phase, and swing phase. All correlations were statistically significant (*p* < 0.001).

### 3.3. Postrehabilitation Gait Modifications

In this study, we compared the MGS and WGS scale measurements before and after rehabilitation treatment for seven participants, with each score representing the average measurements taken by two observers.  Table 3 displays these results. Statistical analysis using the Wilcoxon test showed significant changes in the measurements on both the MGS and WGS scales, with a *p* value of 0.018 for each, highlighting the ability of both scales to identify changes in the gait conditions of participants from T0 to T1.

### 3.4. Healthy Versus Stroke Participant Analysis

Analysis using the Wilcoxon test revealed significant differences between the assessments of the eight healthy participants and the 34 stroke patients (*p* < 0.001). These results indicate that the MGS can effectively differentiate between healthy individuals and those affected by stroke.

### 3.5. Time Efficiency

The average administration times were 7 min and 29 s for MGS, 27 min and 46 s for WGS, and 50 min and 6 s for GAIT. The coefficients of variation were 0.30 for MGS, 0.32 for WGS, and 0.30 for GAIT, demonstrating the relative dispersion of administration times among the scales.

### 3.6. Impact of Illustrations on MGS Usability

Interrater reliability, measured by Cohen's *κ*, showed high agreement for both versions when considering total scores: 0.882 for the illustrated version and 0.862 for the text-only format. The Wilcoxon test did not reveal significant differences in assessment speed (*p* = 0.166), with average speeds of 7 min and 55 s for the illustrated version and 8 min and 48 s for the text-only version. The coefficients of variation for the assessment times were both 0.27, indicating similar variability in assessment duration across both versions.

## 4. Discussion

The MGS proved to be a reliable tool capable of measuring gait alterations on par with the WGS and the GAIT. Despite the historically reduced reliability often associated with OGA, the results from all three scales examined in this study demonstrated consistently almost perfect agreement (*κ* between 0.81 and 1.00) in both interrater and intrarater reliability for the total scores.

These assessments were conducted by professionals with diverse levels of experience and no prior familiarity with the scales. One of the reasons for the robust agreement in measurements could be the introduction of a standardized video recording protocol applied across all scales, which likely contributed to uniformity in observations. However, the impact of recording standards on the reliability of OGA remains an area that warrants further exploration in subsequent studies.

Among the individual items of the MGS, the “foot direction” and “hip hiking” items recorded the lowest levels of agreement, potentially indicating specific challenges inherent to these measurements. The “foot direction” item, which necessitates continuous observation of multiple body segments throughout a gait subphase, yielded lower reliability scores (interrater: 0.760, intrarater: 0.856). This might be due to the complexity of tracking dynamic limb movements over time, which could affect the consistency of evaluations.

Similarly, the “hip hiking” item, with *κ* values of 0.707 for interrater and 0.921 for intrarater reliability, suggests that continuous assessment of hip motion throughout the entire initial swing subphase may also pose challenges. It is conceivable that the requirement to monitor a single element over an extended movement phase complicates accurate and consistent measurement.

The correlation analysis shows that the MGS measures gait similarly to established scales such as WGS and GAIT, with significant *p* values (< 0.001) across all correlations. The slight decrease in correlation during the swing phase (WGS: *ρ* = 0.801, GAIT: *ρ* = 0.770), compared to the total scores (WGS: *ρ* = 0.898, GAIT: *ρ* = 0.877) and the stance phase (WGS: *ρ* = 0.867, GAIT: *ρ* = 0.801), may be attributed to the dynamic nature of the items evaluating gait deviations in this phase.

The MGS demonstrates substantial time efficiency in gait assessment, requiring only 7 min and 29 s on average, significantly less than 27 min and 46 s for the WGS and 50 min and 6 s for the GAIT, with statistical significance supported by the Wilcoxon test results (*p* < 0.001). This efficiency is likely attributable to the smaller number of assessment items: seven for MGS compared to 14 for WGS and 31 for GAIT and the need for analysis solely from the sagittal view, whereas both the WGS and GAIT require video analysis from three different views. Although the actual data collection time was not measured, the requirement to record only a single video for MGS, as opposed to three for the other scales, could significantly contribute to the overall efficiency of the scale.

The evaluation of the impact of images on interrater agreement demonstrated that both the image-based and text-only formats of the MGS exhibited almost perfect agreement, with *κ* values of 0.882 and 0.862, respectively. This indicates that the presence of images has a negligible impact on interrater agreement. The drawing format showed a time reduction, with assessments completed in 7 min and 55 s compared to 8 min and 48 s for the written format, representing a 10% reduction in time. Despite this, the CV was consistent at 0.27 for both formats, indicating stable efficiency. The difference in time efficiency between the formats was not statistically significant (*p* = 0.166). Given the small sample size of 12 stroke patients and 16 raters, further research is necessary to robustly evaluate the impact of images on scale efficiency.

In the rehabilitation setting, the decision to implement an image-based scale, along with a scoring system that ranges up to 100 to indicate the maximum level of recovery, arose from the need for professionals to effectively communicate results and areas of improvement to patients. A scale where 100 represents the highest level of gait ability is likely more comprehensible for patients, as it aligns with familiar concepts of grading or percentages (e.g., achieving 100% indicates complete recovery). We propose that a better understanding of their progress will likely increase adherence to rehabilitation programs among patients and strengthen the therapeutic alliance with their rehabilitation team [27]. While these drawings are intended to simplify the explanation of complex gait assessments and the scoring range is designed to enhance communication clarity, the actual impact of these elements on adherence and outcomes among patients remains to be verified by further research.

## 5. Conclusions

In conclusion, our study validates the MGS as an effective tool for assessing gait in stroke survivors. This concise, 7-item scale provides a streamlined evaluation process, suitable for the time constraints of clinical practice. Demonstrating high reliability and validity, the MGS is comparable to more complex scales such as the WGS and the GAIT, yet it requires significantly less administration time. Notably, the MGS employs just a common smartphone for video recording, enhancing its ease of use. This feature enables physical therapists to perform assessments more frequently, which is essential for monitoring treatment effectiveness and guiding therapeutic decisions.

## Figures and Tables

**Table 1:** Weighted Cohen's kappa (*κ*) values for interrater and intrarater reliability of the MGS items. The values indicate the level of agreement between raters. Interrater reliability and intrarater reliability are provided with their respective 95% asymptotic confidence intervals.

**Item**	**Interrater weighted ** **κ**	**95% asymptotic confidence interval**	**Intrarater weighted ** **κ**	**95% asymptotic confidence interval**
1. Support	0.956	0.907–1.000	0.989	0.978–1.000
2. Contact area	0.803	0.671–0.933	0.943	0.903–0.982
3. Arm swing	0.868	0.764–0.972	0.960	0.931–0.990
4. Joint relations	0.786	0.643–0.929	0.940	0.901–0.978
5. Knee flexion	0.851	0.748–0.954	0.913	0.849–0.976
6. Hip hiking	0.707	0.551–0.863	0.921	0.858–0.984
7. Foot direction	0.760	0.623–0.896	0.856	0.779–0.932
Total scores	0.952	0.921–0.982	0.977	0.966–0.988

**Table 2:** Spearman's correlation coefficients (*ρ*) between the MGS and the WGS and GAIT for total scores, stance phase, and swing phase.

	**MGS total scores**	**MGS stance phase**	**MGS swing phase**
WGS	0.898	0.867	0.801
GAIT	0.877	0.801	0.770

**Table 3:** Comparison of MGS and WGS scale measurements before (T0) and after (T1) rehabilitation treatment for seven participants.

**Participant**	**MGS T0**	**MGS T1**	**WGS T0**	**WGS T1**
4	38.00	57.00	35.25	27.00
21	93.00	97.50	19.35	13.35
25	82.00	95.00	21.23	13.35
34	69.00	76.00	24.90	20.60
41	46.00	63.00	28.30	23.70
42	54.50	62.50	29.68	26.18
45	48.00	56.00	25.30	23.80

*Note:* T0 and T1 values represent the average total scores from two observers. Statistics: Wilcoxon's test for differences in measurements. MGS *p* value: 0.018. WGS *p* value: 0.018.

## Data Availability

The data that support the findings of this study are openly available in Malta Gait Scale Validation at 10.5281/zenodo.11317215.

## References

[1] Kuriakose D., Xiao Z. (2020). Pathophysiology and treatment of stroke: present status and future perspectives. *International Journal of Molecular Sciences*.

[2] Benjamin E. J., Blaha M. J., Chiuve S. E. (2017). Heart disease and stroke statistics—2017 update: a report from the American Heart Association. *Circulation*.

[3] Katan M., Luft A. (2018). Global burden of stroke. *Seminars in Neurology*.

[4] Langhorne P., Coupar F., Pollock A. (2009). Motor recovery after stroke: a systematic review. *The Lancet Neurology*.

[5] Langhorne P., Bernhardt J., Kwakkel G. (2011). Stroke rehabilitation. *The Lancet*.

[6] Timmermans C., Roerdink M., van Ooijen M. W., Meskers C. G., Janssen T. W., Beek P. J. (2016). Walking adaptability therapy after stroke: study protocol for a randomized controlled trial. *Trials*.

[7] Li S., Francisco G. E., Zhou P. (2018). Post-stroke hemiplegic gait: new perspective and insights. *Frontiers in Physiology*.

[8] Mansfield A., Inness E. L., McIlroy W. E. (2018). Stroke. *Handbook of Clinical Neurology*.

[9] Ilunga Tshiswaka D., Bennett C., Franklin C. (2018). Effects of walking trainings on walking function among stroke survivors: a systematic review. *International Journal of Rehabilitation Research*.

[10] Mercier L., Audet T., Hébert R., Rochette A., Dubois M.-F. (2001). Impact of motor, cognitive, and perceptual disorders on ability to perform activities of daily living after stroke. *Stroke*.

[11] Prvu Bettger J. A., Coster W. J., Latham N. K., Keysor J. J. (2008). Analyzing change in recovery patterns in the year after acute hospitalization. *Archives of Physical Medicine and Rehabilitation*.

[12] Ferrarello F., Bianchi V. A. M., Baccini M. (2013). Tools for observational gait analysis in patients with stroke: a systematic review. *Physical Therapy*.

[13] Toro B., Nester C. J., Farren P. C. (2003). The status of gait assessment among physiotherapists in the United Kingdom11No commercial party having a direct financial interest in the results of the research supporting this article has or will confer a benefit upon the authors(s) or upon any organization with which the author(s) is/are associated. *Archives of Physical Medicine and Rehabilitation*.

[14] Toro B., Nester C. J., Farren P. (2003). A review of observational gait assessment in clinical practice. *Physiotherapy Theory and Practice*.

[15] Krebs D. E., Edelstein J. E., Fishman S. (1985). Reliability of observational kinematic gait analysis. *Physical Therapy*.

[16] Ridao-Fernández C., Piñero-Pinto E., Chamorro-Moriana G. (2019). Observational gait assessment scales in patients with walking disorders: systematic review. *BioMed Research International*.

[17] Turani N., Kemiksizoğlu A., Karataş M., Özker R. (2004). Assessment of hemiplegic gait using the Wisconsin Gait Scale. *Scandinavian Journal of Caring Sciences*.

[18] Wellmon R., Degano A., Rubertone J. A., Campbell S., Russo K. A. (2015). Interrater and intrarater reliability and minimal detectable change of the Wisconsin Gait Scale when used to examine videotaped gait in individuals post-stroke. *Physiotherapy*.

[19] Brunnekreef J. J., van Uden C. J. T., van Moorsel S., Kooloos J. G. M. (2005). Reliability of videotaped observational gait analysis in patients with orthopedic impairments. *BMC Musculoskeletal Disorders*.

[20] Rodriquez A. A., Black P. O., Kile K. A. (1996). Gait training efficacy using a home-based practice model in chronic hemiplegia. *Archives of Physical Medicine and Rehabilitation*.

[21] Daly J. J., Nethery J., McCabe J. P. (2009). Development and testing of the Gait Assessment and Intervention Tool (G.A.I.T.): a measure of coordinated gait components. *Journal of Neuroscience Methods*.

[22] Lu X., Hu N., Deng S., Li J., Qi S., Bi S. (2015). The reliability, validity and correlation of two observational gait scales assessed by video tape for Chinese subjects with hemiplegia. *Journal of Physical Therapy Science*.

[23] Yaliman A., Kesiktas N., Ozkaya M., Eskiyurt N., Erkan O., Yilmaz E. (2014). Evaluation of intrarater and interrater reliability of the Wisconsin Gait Scale with using the video taped stroke patients in a Turkish sample. *NeuroRehabilitation*.

[24] Sim J., Wright C. C. (2005). The kappa statistic in reliability studies: use, interpretation, and sample size requirements. *Physical Therapy*.

[25] Landis J. R., Koch G. G. (1977). The measurement of observer agreement for categorical data. *Biometrics*.

[26] Mukaka M. M. (2012). Statistics corner: a guide to appropriate use of correlation coefficient in medical research. *Malawi Medical Journal*.

[27] Atreja A., Bellam N., Levy S. R. (2005). Strategies to enhance patient adherence: making it simple. *Medscape General Medicine*.

